# Solution Structure of the Circular γ-Domain Analog from the Wheat Metallothionein E_c_-1

**DOI:** 10.3390/molecules181114414

**Published:** 2013-11-21

**Authors:** Katsiaryna Tarasava, Silke Johannsen, Eva Freisinger

**Affiliations:** Institute of Inorganic Chemistry, University of Zurich, Winterthurerstrasse 190, Zurich CH-8057, Switzerland

**Keywords:** plant metallothioneins, metal-thiolate cluster, backbone cyclization, flexibility reduction, NMR spectroscopy

## Abstract

The first cyclic analog of a metallothionein (MT) was prepared and analyzed by UV and (magnetic) circular dichroism spectroscopy, ESI-MS as well as NMR spectroscopy. Results reveal that the evaluated cyclic γ-E_c_-1 domain of the wheat MT E_c_-1 retains its ability to coordinate two Zn(II) or Cd(II) ions and adopts a three-dimensional structure that is highly similar to the one of the linear wild-type form. However, the reduced flexibility of the protein backbone facilitates structure solution significantly and results in a certain stabilization of metal binding to the protein.

## 1. Introduction

Metallothioneins (MTs) are low molecular mass proteins with extraordinarily high cysteine (Cys) contents that allow them to coordinate multiple metal ions with d^10^ electron configuration in form of metal-thiolate clusters. MTs are widespread in almost all phyla of life. They are proposed to play a specific role in storage and transport of the essential metal ions Zn(II) and Cu(I) and to participate in the detoxification of physiologically harmful metal ions such as Hg(II) and Cd(II). In addition, the high thiol contents of these proteins and their induction by certain cellular stress conditions suggest a function in the direct scavenging of cell damaging reactive oxygen species, *i.e.*, HO^•^ and O^2•^, resulting in disulfide bridge formation of the Cys residues [1–7].

Metal ion coordination goes along with the deprotonation of the Cys thiol groups. While the p*K*_a_ value of free Cys is around 8.3, the presence of Zn(II) and Cd(II) ions reduces this value by four to five log units and accordingly enables metal-thiolate cluster formation already at slightly acidic conditions and leads to thermodynamically highly stable complexes. So far, three cluster types formed with divalent metal ions, *i.e.*, Zn(II) and Cd(II), have been structurally characterized: The α-cluster with the stoichiometric composition M(II)_4_Cys_11_ is found in vertebrate and echinodermata MTs [8–12], the β-cluster, M(II)_3_Cys_9_, forms the second domain in all vertebrate MTs and also occurs in the crustacean forms [8,9,11,12], while the γ-cluster, M(II)_2_Cys_6_, which is the subject of this paper, was so far only identified in one of the two domains of the E_c_-1 protein, which is very abundant in seeds of *Triticum aestivum* (common bread wheat) [13]. All three cluster types are highly organized, with all Cys residues of the protein sequence participating in metal ion coordination. In addition, the Zn(II)- and Cd(II)-forms of the clusters are usually isostructural. MTs contain generally very little or even no secondary structural elements such as α-helices or β-sheets and consequently their structure and folding is entirely dictated by the coordination of metal ions and the cluster structures formed. The metal-free apo-forms are largely unfolded.

The plant MTs are divided into four subgroups mostly according to the number and distribution of the Cys residues. The members of these four subgroups are denoted as MT1, MT2, MT3, and E_c_-1 proteins [14]. While all plant MTs usually contain two Cys-rich regions, the E_c_-1 proteins contain a third Cys-rich stretch and in addition also two highly conserved histidine residues [2,3]. The E_c_-1 protein from wheat embryos was the first MT identified in higher plants [15]. It is a naturally Zn(II) containing protein and features two metal binding domains, the N-terminal γ-domain that contains the above mentioned γ-cluster and the β_E_-domain, or extended β-domain, formed by the central and C-terminal Cys-rich region including the two highly conserved histidine residues [16]. The β_E_-domain hosts a cluster with the composition Zn_3_Cys_9_ as observed in the β-cluster from vertebrate MTs as well as a mononuclear Zn(II)Cys_2_His_2_ binding site, which is similar to those observed in certain Zn-finger proteins but unique for MTs so far [17]. Also the structure of the γ-E_c_-1 cluster, determined previously by us with NMR spectroscopy [13], is not new as such but can be found in yeast transcription factors, e.g., in GAL4 [18]. Such a two-metal ion cluster, however, is again absolutely unique for MTs. As in GAL4, the three-dimensional structure reveals coordination of the two divalent metal ions by four terminal and two bridging Cys residues, and accordingly represents the smallest metal-thiolate cluster possible. The second bridging Cys residue could not be assigned unambiguously in the wild-type structure, and [^113^Cd,^1^H]-HSQC experiments performed with ^113^Cd_2_γ-E_c_-1 were in line with two alternative cluster arrangements [13]. The encountered difficulty in structure assignment can be due to some dynamic behavior of the protein, which might be important for the proper function of the protein. However, restriction of protein flexibility might lead to the stabilization of one conformation and hence facilitate a more detailed structural characterization of the γ-E_c_-1 domain.

One way to reduce the flexibility of a protein is backbone cyclization. Such circular proteins and peptides occur also *in vivo*. Among the most prominent representatives are the cyclotides, which probably have a role in plant defense mechanisms against insect pests or certain microbes [19]. In these proteins, backbone cyclization in combination with multiple disulfide bridges also increases the stability against heat denaturation and proteolytic digestion [20,21]. *In vitro* studies confirmed that the absence of free N- and C-termini confers protection against degradation by peptidases, increases the thermal stability, and additionally leads to an exceptional physicochemical stability of the circular compound compared to the corresponding linear analog [22,23].

Herein we describe the three-dimensional structure and metal ion binding properties of the recombinantly produced backbone cyclized γ-domain of the wheat MT E_c_-1, *i.e.*, cyc-γ-E_c_-1. To our knowledge, this has never been attempted for any MT so far. We can show that the influence of backbone cyclization on the structure is minor, while a slight affinity increase for Zn(II) and Cd(II) can be observed.

## 2. Results and Discussion

### 2.1. Design, Purity, and Metal Ion Binding Abilities of the Circular γ-E_c_-1 Analog

**Figure 1:**
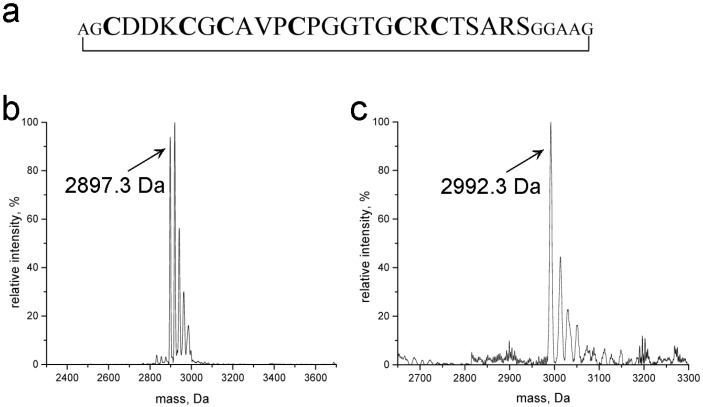
Sequence and purity of cyc-γ-E_c_-1. (**a**) Amino acid sequence of cyc-γ-E_c_-1. The sequence of the wild-type linear form is given in capital letters, Cys residues are highlighted with bold letters, amino acids of the additional linker region are given in lower case letters. (**b**) Deconvoluted ESI-MS spectrum of cyc-Zn_2_γ-E_c_-1 (calculated mass of molecular ion 2,897.9 Da). (**c**) Deconvoluted ESI-MS spectrum of cyc-Cd_2_γ-E_c_-1 (calculated mass of molecular ion 2,991.9 Da). Peaks with a higher mass correspond to the Na-adducts of the respective species.

Backbone cyclization was achieved using an optimized intein-mediated protein ligation approach developed in our lab [24]. In order to ensure efficient joining of the N- and C- termini without introduction of steric strain that might also influence the metal ion coordination capabilities of the protein, a short linker region composed of seven glycine and alanine residues was introduced (Figure 1a). After purification, the metal-free apo-peptide was reconstituted with metal ions and the purity of the Zn^II^- and Cd^II^-loaded forms of cyc-γ-E_c_-1 was confirmed with electrospray ionization mass spectrometry (ESI-MS, Figure 1b,c). In particular, the spectra also reveal the absence of oxidized species, *i.e.*, of disulfide-bridge containing forms, and accordingly, the circular species retained the same stability against oxidation by air as the linear form.

To corroborate the ESI-MS spectra the ratio between bound metal ions and cyc-γ-E_c_-1 was determined analytically. Measurements of the metal ion concentrations with flame atomic absorption spectroscopy (F-AAS) and of the protein concentration via quantification of thiol groups using the 2,2′-dithiodipyridine (2-PDS) assay [25] gave metal ion-to-protein ratios of 2:1 for both Zn^II^ and Cd^II^. In addition, protein dimerization via non-covalent interactions was excluded by size exclusion chromatography (SEC) of cyc-Zn_2_ and cyc-Cd_2_γ-E_c_-1, which produced an elution profile with a single symmetric peak without shoulders, respectively, corresponding to the monomeric form (Figure S1).

**Figure 2:**
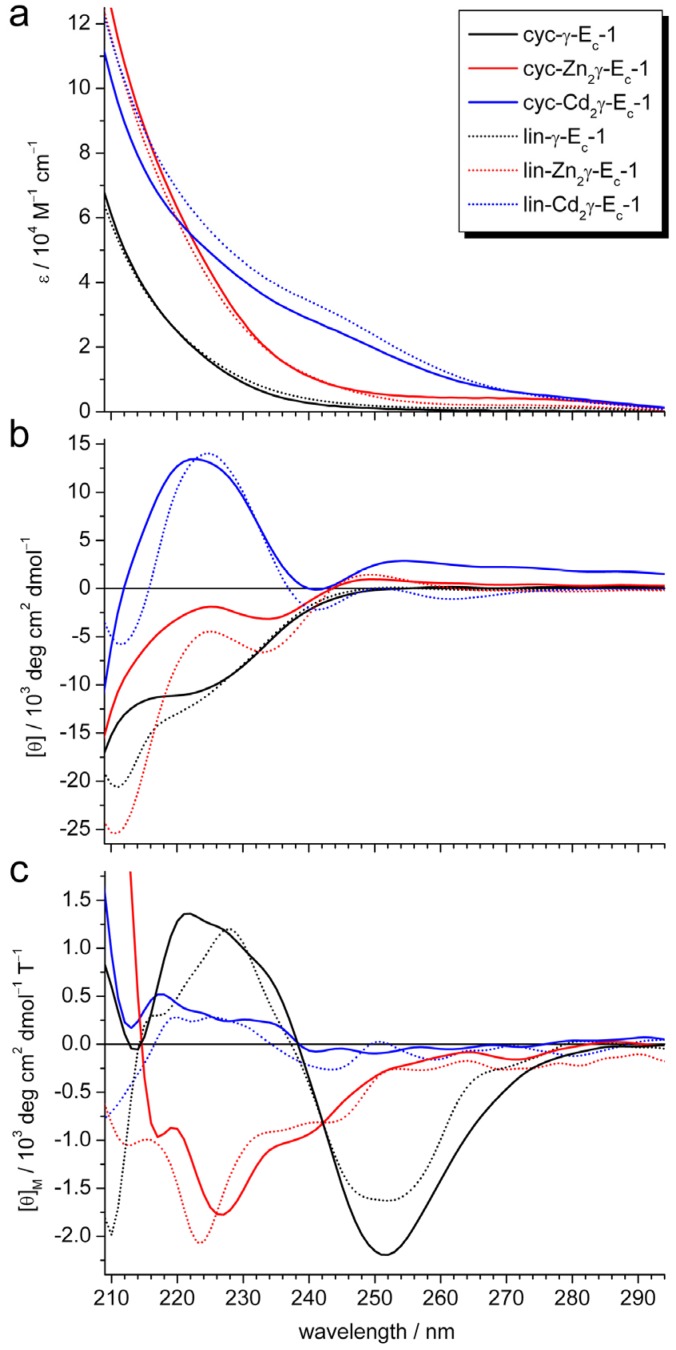
Comparison of (**a**) UV, (**b**) CD, and (**c**) MCD spectra of circular metal-free, Zn_2_-, and Cd_2_γ-E_c_-1 with the respective linear wild-type forms. See legend in (**a**) for assignment of spectra.

Experiments with UV, circular dichroism (CD), and magnetic circular dichroism (MCD) were performed to verify that the two divalent metal ions bound to cyc-γ-E_c_-1 are still coordinated in form of a metal-thiolate cluster as observed for the linear protein domain. Zn^II^ and Cd^II^-thiolate coordination results in typical ligand-to-metal charge transfer (LMCT) bands around 230 and 250 nm, respectively. Comparison of the UV spectra of circular metal-free, Zn_2_-, and Cd_2_γ-E_c_-1 with the respective linear forms reveals similar shapes and also similar extinction coefficients indicating that no major difference in metal-thiolate bond formation occurs due to the backbone cyclization (Figure 2a).

This information is complemented by (M)CD data that can in addition provide information about cluster formation and structural rearrangement processes due to the sensitivity of this method to dipole-dipole interactions [26]. The shape of the CD and MCD spectra of cyc-Zn_2_- and cyc-Cd_2_γ-E_c_-1 (Figure 2b,c) are closely similar to the corresponding spectra of the linear forms. The CD spectra of both Cd^II^-forms reveal extrema around (+)252 and (−)242 nm, and the spectra of the Zn^II^-forms have extrema at (+)250 and (−)235 nm (Figure 2b). Such features have been associated with the formation of clustered structures. This is corroborated by the pronounced minima at (−)252 nm observed in the MCD spectra of both Cd^II^-forms (Figure 2c), which is again indicative for a Cd^II^-thiolate cluster [26].

### 2.2. pH Stability of the Metal-Thiolate Clusters

**Figure 3:**
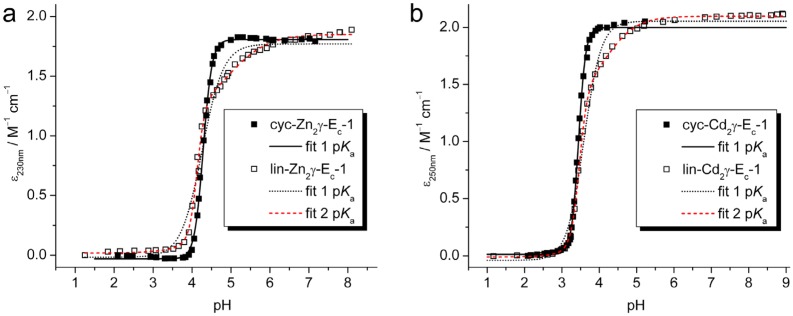
Plots of molar absorptivity against pH values for the pH titrations of (**a**) cyc-Zn_2_- and (**b**) cyc-Cd_2_γ-E_c_-1 and the respective linear forms including the data fits using Equation (1) or (2) (see Table 1). For assignment of data and curves see the legends.

To compare the stabilities of the metal-thiolate clusters in cyc- and linear γ-E_c_-1 pH titrations were performed. For this, the metal-bound sample is stepwise acidified and changes of the LMCT band at 230 or 250 nm, respectively, are monitored with UV spectroscopy. The absorptivity decrease of the LMCT bands directly represents metal ion release from the clusters. Plots of the respective extinction coefficients in dependence of the pH value of the solution are depicted in Figure 3.

To obtain the apparent p*K*_a_ values of the Cys residues in the presence of the respective metal ions, fitting of the plots with an equation considering the presence of just a single p*K*_a_ value for all Cys residues of the protein can be performed. The results are given in Table 1 and reveal that the p*K*_a_ values determined for the two Zn^II^-forms are equal within the 3σ level, while the p*K*_a_ value for cyc-Cd_2_γ-E_c_-1 is slightly lower than the one for the linear form. Apparent though are the distinctively different values for *n*, *i.e.*, 3.9(1) and 4.5(1) for the two circular forms compared to 1.6(1) and 1.9(1) for the respective linear forms. The coefficient *n* in Equation (1) (Table 1) is similar to the Hill coefficient in the respective equation [27], however it does not indicate cooperativity of metal ion coordination but rather is a measure for the amount of Cys residues in a structure with closely similar p*K*_a_ values without giving the precise number. In other words, in the two cyclic forms all six thiolate groups are approximately protonated at the same pH, and hence also both metal ions, *i.e.*, two Zn^II^ or two Cd^II^ ions, are roughly released at once around pH 4.3 and 3.4, respectively. In contrast, the complexes of linear wild-type E_c_-1 show a metal ion release process occurring in two steps. This can be shown very clearly when the respective plots are fitted with an equation that considers two independent p*K*_a_ values (Equation (2), Table 1). The first deprotonation step at higher pH is accompanied by a decrease of the absorptivity of roughly 30% in both cases, corresponding to the protonation of two of the six thiolate ligands. Accordingly the higher p*K*_a_ values mark the release of the first Zn^II^ or Cd^II^ ion while the two lower p*K*_a_ values indicate release of the second metal ion and hence protonation of the other four thiolate groups. Accordingly, the major difference between the cyclic and the linear forms revealed by the pH titration studies is the uniform pH stability of the two metal ion binding sites in the cyclic forms, while the linear forms show sequential metal ion release. In other words, cyclization stabilizes the less stable metal binding site observed in the wild-type γ-domain.

**Table 1:** Apparent p*K*_a_ values of Cys residues in different γ-E_c_-1 species obtained by fitting of pH titration data using two different equations ^[a,b]^.

Equation 1	cyc-Zn_2_γ-E_c_-1	lin-Zn_2_γ-E_c_-1	cyc-Cd_2_γ-E_c_-1	lin-Cd_2_γ-E_c_-1
A_MT_	18062 ± 67	17696 ± 261	19981 ± 109	20539 ± 200
A_MTHn_	−295 ± 82	−161 ± 372	116 ± 101	−395 ± 361
p*K*	4.287 ± 0.003	4.26 ± 0.03	3.444 ± 0.003	3.59 ± 0.02
*n*	3.85 ± 0.10	1.6 ± 0.1	4.5 ± 0.1	1.9 ± 0.1
**Equation 2**				
A_MT_		18504 ± 141		20979 ± 61
A _MTHm_		11518 ± 1061		13634 ± 1174
A _MTHn+m_		176 ± 172		−109 ± 124
p*K*_1_		4.129 ± 0.010		3.466 ± 0.007
p*K*_2_		4.9 ± 0.2		4.2 ± 0.1
*n*		4.3 ± 0.5		3.9 ± 0.3
*m*		0.9 ± 0.1		1.1 ± 0.1

^[a]^ 
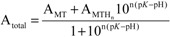
 (1); ^[b]^ 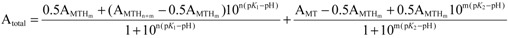 (2).

A_MT_ is the absorptivity of the fully metal ion-loaded protein (=A_max_), A_MTHn_ (Equation (1)) and MTH_n+m_ (Equation (2)) denote the value obtained for apo-MT after acidification (=A_min_), MTH_m_ (Equation (2)) is the absorptivity of the protein species obtained after the first protonation step characterized by p*K*_2_, and m (Equation (2)) as well as n (Equations (1) and (2)) are a measure for the slope of the curves.

### 2.3. Structural Analysis with NMR Spectroscopy

#### 2.3.1. Conformational Constraints and Structure Calculation

The solution structure of cyc-Cd_2_γ-E_c_-1 was determined by NMR spectroscopy to analyze the influence of cyclization on the overall structure and cluster arrangement of the domain. The assignments are complete for the range G2-R25, which are mostly the amino acids of the wild-type sequence that are wrapped around the cluster structure. However, only very few protons for the residues of the linker region (S26-G31) can be assigned, in particular almost no inter-residues NOE connectivities were found making structure solution of this region impossible.

P14 is connected via a trans-peptide bond to the previous Cys residue as observed in the linear domain according to an NOE found between the Pro H^δ^^3^ proton and Cys H^N^ (Bruker Biospin NMR Guide 3.5 [28]). For P12 no spectral evidence for a cis or trans conformation was observed due to overlapping signals with the water line. However, the results of the structure calculations reveal significantly lower energies for a cis-bond to the previous Val (Table S1). To improve structure quality and to bring the model closer to the native protein structure further refinement in explicit solvent was performed (for further details see Experimental Section) [29].

**Figure 4:**
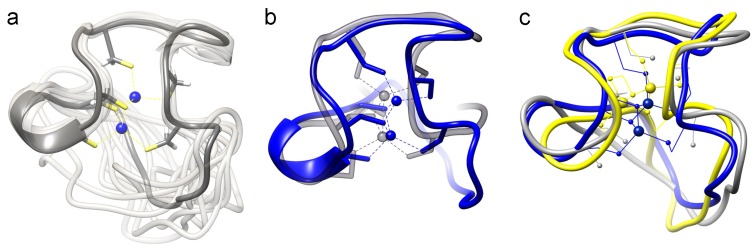
NMR solution structure of cyc-γ-E_c_-1. (**a**) Protein backbones of the 20 lowest energy structures of cyc-Cd_2_γ-E_c_-1 in ribbon presentation. The structure closest to the average structure is depicted in dark grey and also the Cd^II^-thiolate cluster arrangement is shown (Cd^II^ ions as blue spheres, Cys thiolates as yellow sticks); (**b**) Backbone overlay of cyc- (blue) and linear (grey) Cd_2_γ-E_c_-1 structures that are closest to the respective mean structure (Cd^II^ ions as spheres, Cd^II^-thiolate connectivities are indicated with dotted lines); (**c**) Protein backbone overlays of cyc-Cd_2_γ-E_c_-1 with Cys3 and Cys9 (blue) or Cys21 and Cys9 (yellow) as bridging residues between the two Cd^II^ ions of the cluster and the structure that was calculated without any metal restraints (grey). Cd^II^ ions are shown as larger spheres and the thiolate groups of the Cys residues as smaller spheres.

Superposition of the 20 structures with the lowest energy and best values of the ø, ψ angles (Figure 4a) yields a total RMSD of 0.80 Å for the backbone, metal cluster and Cys-Cβ atoms of the non-linker residues, *i.e.*, residues 2-23. The RMSD for all non-linker heavy atoms, *i.e.*, residues 1-24, is 1.24 Å. The statistics for the structure bundle are presented in Table 2. Validation of the structures with PROCHECK-NMR [30] reveals that 100% of the ø, ψ angles for the best 20 structures are in the core of allowed regions after refinement in explicit solvent. No NOE violations > 0.3 Å and RMS values for bond deviations from ideality > 0.016 Å or RMS values for angle deviations from ideality > 1.4 were observed. The NMR solution structure of cyc-Cd_2_γ-E_c_-1 was deposited in the Protein Data Bank under accession number 2MFP.

**Table 2:** Statistics of the 20 lowest energy structures before and after refinement in explicit solvent.

*NOE restraints*		
Total	201	
Sequential (|i-j| < 1)	142	
Medium (1 < |i-j| <5)	38	
Long range (|i-j| <5)	21	
RMSD to average structure, Å (res. 2-23) Before water refinement		After water refinement
Backbone (N,C_α_,C)	0.71	0.80
All heavy atoms	1.03	1.10
Number of close contacts(within 1.6 Å for H atoms, 2.2 Å for heavy atoms)	18	0
RMS deviation for bond angles (°)	0.2	1.4
RMS deviation for bond lengths(Å)	0.001	0.016
Ordered residues	3A-14A	2A-23A
Procheck-NMR (residues 2-23) [30]		
In most favored regions (%)	58,3	73.9
In additional allowed (%)	41.7	26.1
In generously allowed (%)	0	0
In disallowed regions (%)	0	0
Verify3D (residues 2-23) [31]		
Raw score	0.43	0.5
Z-score	−0.48	0.64

#### 2.3.2. Comparison of the cyc-γ-Ec-1 Structure with its Linear Analog

**Figure 5:**
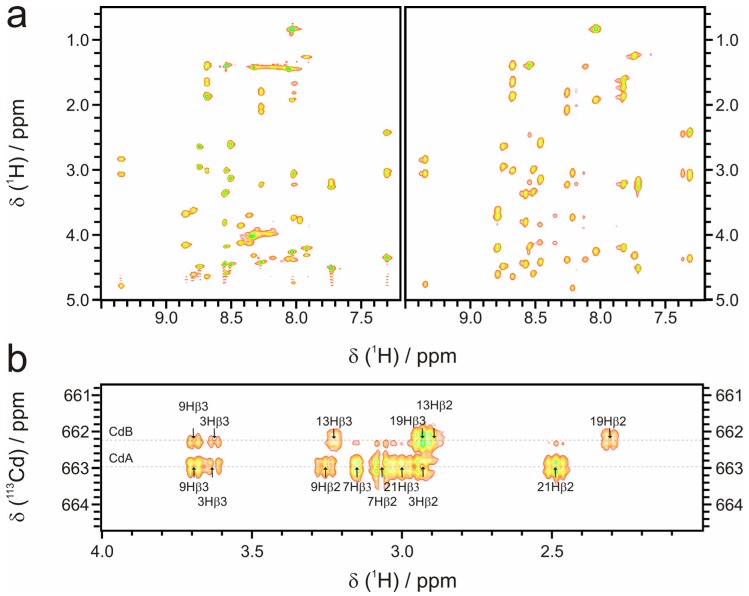
Selected NMR spectra of Cd_2_γ-E_c_-1. (**a**) Comparison of the amide regions of the 2D [^1^H,^1^H] TOCSY NMR spectra of cyc- (left) and linear (right) Cd_2_γ-E_c_-1. Evidently, most of the resonances have similar chemical shifts in the two species; (**b**) [^113^Cd,^1^H]-HSQC spectra of cyc-Cd_2_γ-E_c_-1 including cross peak assignments.

Judging from the spectrophotometric data (Figure 2) no distinct differences of the structural arrangement in the linear and circular γ-E_c_-1 domain are expected. This is corroborated by the detailed analyses with NMR spectroscopy. For example, the amide regions of the 2D [^1^H,^1^H]-TOCSY spectra overlay almost completely (Figure 5a) and the comparison of backbone amide ^N^H and ^α^H chemical shifts of the residues from the well resolved regions of the circular and linear domain shows mostly only small differences of less than 0.1 ppm. Only in regions which are close to the termini of the linear domain this difference is higher (up to 0.3 ppm, Figure S2). Furthermore ^113^Cd-NMR spectroscopy reveals two ^113^Cd resonances with chemical shifts of 662.2 ppm and 663.0 ppm that strongly corroborates coordination of both Cd^II^ ions in a tetrahedral tetrathiolate environment as also observed for the linear analog, *i.e.*, 659.5 and 660.9 ppm (Figure 5b) [13,32].

The NMR solution structures of cyc-Cd_2_γ-E_c_-1 shows a hook-like arrangement of the protein backbone as observed in the linear γ-E_c_-1 domain. The linker region itself that was inserted with the intention to reduce backbone flexibility cannot be resolved by NMR most likely due to its own flexibility. The superposition of the amino acid backbones in cyc- and linear γ-E_c_-1 demonstrates the high similarity of the overall arrangements of the two polypeptide chains around the metal-thiolate clusters as expected (Figure 4b). However, two main differences are observed, most likely originating from the reduced flexibility of the protein backbone in the circular form. The first difference concerns the metal-thiolate cluster. In the linear γ-E_c_-1 domain only one of the bridging Cys ligands, *i.e.* Cys9, was assigned unambiguously based on the [^113^Cd,^1^H]-HSQC spectra. For the second bridging Cys residue Cys3 and Cys21 equally well fit the data. The comparison of the structure calculations for the linear γ-E_c_-1 domain with Cys3 and Cys9 or Cys21 and Cys9 as bridging thiolates reveals RMSD differences of less than 0.01 Å and equal total amber energies within the error limit. Accordingly, a highly dynamic cluster switching between the two slightly different cluster arrangements was suggested [13].

In contrast, the [^113^Cd,^1^H]-HSQC spectra of cyc-Cd_2_γ-E_c_-1 allow the unambiguous assignment of all Cd-H_β_ cross peaks (Figure 5b) and reveal only one possible cluster conformation with Cys3 and Cys9 as bridging residues. To further verify this observation we performed additional structure calculations with Cys3/Cys9 or Cys21/Cys9 as bridging ligands (for details see Experimental section). The quality of peak assignment in CYANA is the same for both cluster arrangements within the error limits, however the number of used NOE restraints to accomplish the structure was significantly higher with Cys3 and Cys9 as bridging ligands. Moreover, the overall structure energies in XPLOR are distinctively lower for structures with Cys3 and Cys9 as bridging thiolates (Table S1). This data that is well in line with the spectrophotometric results above reliably shows that cyclization of the γ-E_c_-1 domain remarkably decreases the flexibility of its metal-thiolate cluster.

**Figure 6:**
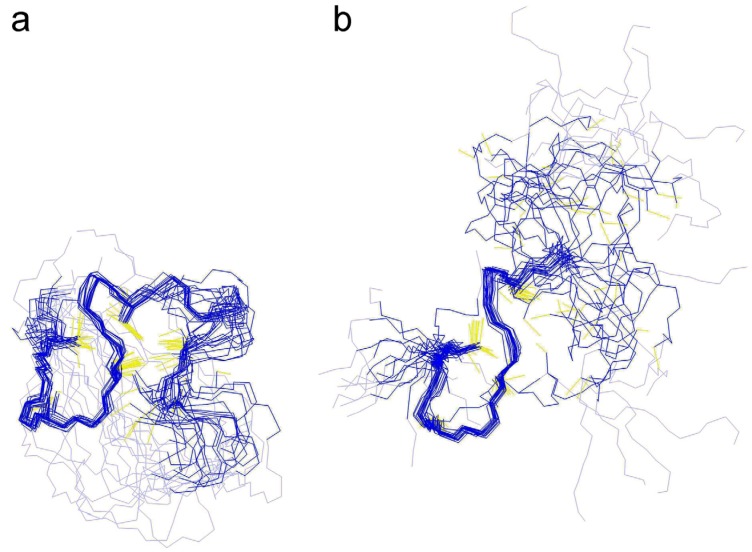
Structure bundles of the 20 lower energy structures of (**a**) cyc- and (**b**) linear Cd_2_γ-E_c_-1 calculated without any metal restraints.

The second difference between the structures concerns the necessity of using cluster restraints for structure determination. If present at all, MTs have generally a very low content of secondary structural elements such as α-helices and β-sheets and accordingly, their structure is governed by the formation of the metal-thiolate clusters. This means that application of metal cluster restraints is usually critical for structure determination and for obtaining a reliable structure. Nevertheless, structure calculation attempts for cyc-Cd_2_γ-E_c_-1 that did not consider any cluster restraints such as ^113^Cd-Cys connectivities or Cd-S distances already gave a structural fold with high similarity to the final structure (Figure 6a), while for the linear domain, such an approach yielded only a poorly defined highly flexible structure, in which only the N-terminal region could be resolved (Figure 6b). In addition for the circular domain even the positions of the bridging Cys residues are very close to the positions of the respective residues in the structure that was calculated with the metal-thiolate restraints. Hence, at least for the γ-E_c_-1 domain, cyclization of the structure allows deduction of the general cluster structure solely based on the orientation of the Cys residues without additional knowledge of metal-thiolate restraints. That fairly good structure determination is possible even without using any metal to cysteine restraints can be visualized by the peptide backbone overlays depicted in Figure 4c. Superposition of the backbone atoms of the structure calculated without metal restraints with the two structures calculated with the assumption that Cys3 and Cys9 or Cys21 and Cys9 are the bridging thiolate groups reveals very similar arrangements. However, to truly distinguish between the two very similar cluster arrangements, *i.e.*, Cys3/Cys9 or Cys21 /Cys3 as bridging ligands, the exact Cd-Cys connectivities based on [^113^Cd,^1^H]-HSQC spectra are still required. The observed differences between cyc- and linear γ-E_c_-1 in the process of structure determination without cluster restraints is mainly due to the distinctively different numbers of long-range NOEs observed (Table S2). Lacking regular secondary structural elements, structure solution of MTs relies heavily on long-range NOEs to precisely define the structure, and accordingly the increased number of long-range NOEs for the circular form compared to the linear wild-type domain strongly facilitates determination of the structure in the absence of any metal to cysteine restraints.

## 3. Experimental

### 3.1. Chemicals and Solutions

^113^CdCl_2_ was purchased from Cambridge Isotope Laboratories (ReseaChem GmbH, Burgdorf, Switzerland) and *d*_11_-tris(hydroxymethyl)aminomethan (*d*_11_-Tris) from Euriso-top (Saint-Aubin, France). Chelex^®^ 100 resin was obtained from Bio-Rad (Reinach, Switzerland). All other chemicals were ACS grade or comparable and purchased from either Sigma-Aldrich (Buchs, Switzerland), Merck (Darmstadt, Germany), Chemie Brunschwig (Basel, Switzerland), or Roth AG (Arlesheim, Switzerland). All solution were prepared using ultrapure water (TKA GenPure, Niederelbert, Germany). When necessary, water was vacuum-degassed and nitrogen-saturated.

### 3.2. Expression and Purification of cyc-γ-E_c_-1

cyc-γ-E_c_-1 was recombinantly expressed without ^15^N or ^13^C enrichment and purified using the modified pTWIN2 vector of the IMPACT^TM^‑CN system (New England Biolabs, Ipswich, MA, USA) as described [24]. The purity of cyc-γ-E_c_-1 and in particular the absence of linear contaminant was confirmed by mass spectrometry.

### 3.3. Preparation of cyc-Zn_2_γ−E_c_-1 and cyc-Cd_2_γ−E_c_-1

Samples of cyc-Zn_2_γ−E_c_-1 and cyc-Cd_2_γ-E_c_-1 were prepared by adding the respective metal ion salt (ZnCl_2_, CdCl_2_ or ^113^CdCl_2_ for the NMR samples) in small excess (2.5–3.0 equiv.) to the apo-form of cyc-γ-E_c_-1 in 10 mM HCl. Subsequently, the pH was raised to 8.0 using 1 M NaOH or 1 M *d*_11_-Tris for the NMR sample. To remove excess metal ions the reconstituted samples were dialyzed against a solution of 10 mM Tris-HCl, pH 7.5, and 10 mM NaCl. For preparation of the NMR samples excess metal ions were removed by dialyzing once against ddH_2_O and two times against 10 mM *d*_11_-Tris-HCl, pH 8.0. Prior to each experiment the protein-to-metal ion ratio was determined. The protein concentration was assessed via quantification of the Cys thiol groups with the 2-PDS assay at pH 4.0 assuming all six cysteine residues to be present in the reduced state [25]. Metal ion concentrations were determined with flame atomic absorption spectroscopy (F-AAS) using an AA240FS spectrometer (Agilent Technologies AG, Basel, Switzerland) in 0.2 M HNO_3_. The calculated metal-to-protein stoichiometries were in good agreement with results from mass spectrometry and from previous experiments [13]. To exclude the possibility of dimer formation cyc-Zn_2_γ−E_c_-1 and cyc-Cd_2_γ-E_c_-1 were applied to a Superdex Peptide HR 10/30 SEC column (GE Healthcare Europe GmbH, Glattbrugg, Switzerland), which was pre-equilibrated with 10 mM Tris-HCl, pH 7.4, and 10 mM NaCl buffer using a flow rate of 0.4 mL min^−1^.

### 3.4. Mass Spectrometry

Samples of the Zn^II^- and Cd^II^-forms of cyc-γ-E_c_-1 were dialyzed against 10 mM NH_4_Ac, pH 7.5, mixed with MeOH (50% final conc.) and injected into a quadrupole time-of-flight (TOF) Ultima API spectrometer (Waters, Milford, MA USA). Scans were accumulated and processed by the software Micromass MassLynx 3.5 (Waters). *m/z* Spectra were deconvoluted using the maximum entropy algorithm (MaxEnt1 in MassLynx 3.5). Electrospray parameters were capillary 2.8 V, cone 60 V and source temperature 80 °C.

### 3.5. UV/Vis, CD, and MCD Spectroscopy

UV/Vis spectra were recorded on a Cary 500 scan spectrophotometer (Agilent Technologies AG, Basel, Switzerland) using a scan speed of 600 nm min^−1^ over the spectral range of 200–500 nm. CD and MCD spectra were acquired on a J-715 spectropolarimeter equipped with a 1.5 T (15 kG) magnet (JASCO, Tokyo, Japan) using a scan speed of 50 nm min^−1^ over the spectral range of 200–300 nm with three spectra accumulations. All spectra were recorded at room temperature with samples containing 20 μM of protein in 10 mM Tris-HCl, pH 7.4, and 10 mM NaCl. 

### 3.6. pH Titrations

20 μM cyc-Zn_2_- or cyc-Cd_2_γ-E_c_-1 in 10 mM Tris-HCl, pH 8.0, and 10 mM NaCl (700 μL) were titrated with small increments of diluted (0.01, 0.1, and 1 M) HCl solutions [16]. Plots of molar absorptivity at 230 nm for cyc-Zn_2_γ-E_c_-1 and at 250 nm for cyc-Cd_2_γ-E_c_-1 *versus* pH were fitted with the program Origin 8.0^®^ (OriginLab, Northampton, MA, USA) considering either a single or two independent apparent p*K*_a_ values for the cysteine residues in the presence of the respective metal ions as described [33].

### 3.7. NMR Spectroscopy

Prior to NMR measurements the sample was lyophilized, re-dissolved in 300 μL H_2_O/D_2_O (90:10), containing 10 mM *d*_11_-Tris-HCl, pH 7.5, and 10 mM NaClO_4_, and transferred into a 5 mm Shigemi^®^ tube (Shigemi Inc., Allison Park, PA, USA). The final concentration of the cyc-^113^Cdγ-E_c_-1 sample was 0.9 mM.

All ^1^H-NMR spectra were recorded on a Bruker AV700 MHz spectrometer equipped with a TXI z-gradient CryoProbe^®^ or on a Bruker AV600 MHz spectrometer equipped with a TCI z-gradient CryoProbe^®^ and the chemical shifts are directly referred to the external standard 4,4-dimethyl-4-silapentane-1-sulfonic acid (DSS, 0.2%, pH 7.5) [34]. ^113^Cd-NMR spectra were recorded on a Bruker DRX500 MHz spectrometer equipped either with a BBI or a BBO probe head. The ^113^Cd chemical shifts are directly referenced to an external 0.1 M Cd(ClO_4_)_2_ solution [35].

The assignment of the proton resonances was performed using 2D NOESY spectra (120 ms, 150 ms, and 250 ms mixing times) and TOCSY spectra (100 ms mixing time) at 298 K and 310 K. Sequence-specific resonance assignment based on the assignment of the linear ^113^Cd_2_γ-E_c_-1 form [13] was performed using the method developed by Wüthrich and coworker [36]. The spin systems were first identified in the TOCSY experiments and then sequentially linked based on NOE information derived from NOESY experiments. To investigate the metal-thiolate cluster 1D ^113^Cd-NMR and [^113^Cd,^1^H]-HSQC spectra were recorded at 298 K and 310 K and subsequently the individual Cd-Cys connectivities were established on the basis of ^3^*J*[H*β*,Cd] couplings deriving from the [^113^Cd,^1^H]-HSQC experiments. All spectra were evaluated using CARA [37], XEASY [38], and Sparky [39], respectively.

### 3.8. Structure Calculation

NOE peaks were integrated with XEASY employing identical lower integration thresholds. To fix the cluster geometry upper and lower distance restraints were added, *i.e.* 3.20 ≤ *d*(Cd, Cd) ≤ 3.60 Å between the metal ions (one restraint), 2.47 ≤ *d*(Cd, S^γ^) ≤ 2.53 Å between a metal ion and its four coordinating Cys residues (eight restraints), and 3.80 ≤ *d*(S^γ^, S^γ^) ≤ 4.40 (eleven restraints) between Cys residues that coordinate the same metal ion. These parameters are derived from an ideally tetrahedral CdCys_4_ geometry with distance ranges chosen to ensure the correct geometry but do not cause structure violations.

For the structure calculations, torsion angle dynamics [40] were performed with the *noeassign* [41] algorithm of the program CYANA 2.1 [42] starting from 500 conformers with randomized torsion angle values. The 100 conformers with the lowest final target function value were selected for restrained energy minimization in explicit solvent against the AMBER force field using the program XPLOR-NIH 2.33 [43,44] as described in [29]. Cd^II^ ions were defined with two positive charges. The Lennard–Jones potential parameters used were sigma = 1.942 Å and eps = 0.01 kcal mol^−1^ [45]. For the water refinement with XPLOR-NIH the tetrahedral geometry of the cluster was fixed using *d*(Cd, S^γ^) = 2.50 Å (with an energy constants of 1,000 kcal mol^−1^ Å^−2^) and for the angles ∢(S^γ^-Cd-S^γ^) = 109° and ∢(Cd-S^γ^-Cd) = 106°, respectively (with energy constants of 500 kcal mol^−1^ rad^−2^).

Structures of cyc-γ-E_c_-1 to compare the influence of different metal-cluster restraints, *i.e.*, Cys3/Cys9 *vs.* Cys21/Cys9 as bridging ligands, and Pro bond formation, *i.e.*, Cys3/Cys9 as bridging ligands and cis or trans P12, were calculated with the *noeassign* algorithm of the program CYANA starting from 100 conformers with randomized torsion angle values. The best 20 structures were further refined using a simple *annealing* protocol in XPLOR-NIH to obtain overall structure energies. All Cd^II^ parameters and angles for the cluster geometry were set as in the *water-refinement* protocol mentioned above. The metal-sulfur connectivities were adapted in dependency of the bridging Cys ligands.

The best 20 structures of cyc- and linear γ-E_c_-1 without any metal restraints were calculated with CYANA starting from 100 conformers in the same way as described above.

For structure validation and visualization the programs PROCHECK-NMR [30] and, respectively, Chimera [46] and Molmol [47] were used.

## 4. Conclusions

In this work we evaluated how cyclization influences the metal binding properties and the structure of the γ-domain from the wheat MT E_c_-1. This well-understood and rather simple system hereby was intended to serve as a general example for a metalloprotein. Using spectroscopic techniques, MS spectrometry, as well as direct analytical methods for the determination of metal to protein contents we showed that in the present example cyclization neither affects the metal ion binding stoichiometry nor disturbs the cluster structure arrangement. The circular γ-E_c_-1 domain is still able to bind two d^1^° metal ions, *i.e.*, Zn(II) or Cd(II), involving all six Cys residues for coordination. In addition, a slight improvement of the pH stability of the second metal ion binding site was observed. Comparison of NMR structures shows similar overall folds in the cyclic and in the linear wild-type domain. However, cyclization apparently reduced the flexibility of the cluster and let to the stabilization of only one of the two possible conformers observed in the linear γ-E_c_-1 domain. Results of this study show the possibility to use N- to C-terminal cyclization for improvement of metal binding abilities and reduction of structure flexibility. This method may be applicable or at least worth exploring for similar cases and particularly for other MTs and MT domains.

## Supplementary Materials

Supplementary materials can be accessed at: http://www.mdpi.com/1420-3049/18/11/14414/s1.
