# Supplementary material for: Solution Structure of the Circular γ-Domain Analog from the Wheat Metallothionein Ec-1
Source: Molecules. 2013 Nov 21;18(11):14414–29. doi: 10.3390/molecules181114414 (PMC6269658; doi:10.3390/molecules181114414)
Supplement: Supplementary file 1 [file molecules-18-14414-s001.pdf]

Supplementary Materials

Figure S1. SEC elution profile of cyc-$Zn_2\gamma$-E_c-1. The single symmetric peak with an elution volume of 12.1 mL corresponds to monomeric cyc-$Zn_2\gamma$-E_c-1. Peaks with elution volumes larger than 17 mL originate from buffer components. Experimental details: Superdex Peptide HR 10/30 column (GE Healthcare), flow rate 0.4 mL min^{-1}, 10 mM Tris-HCl pH 7.4, 10 mM NaCl.

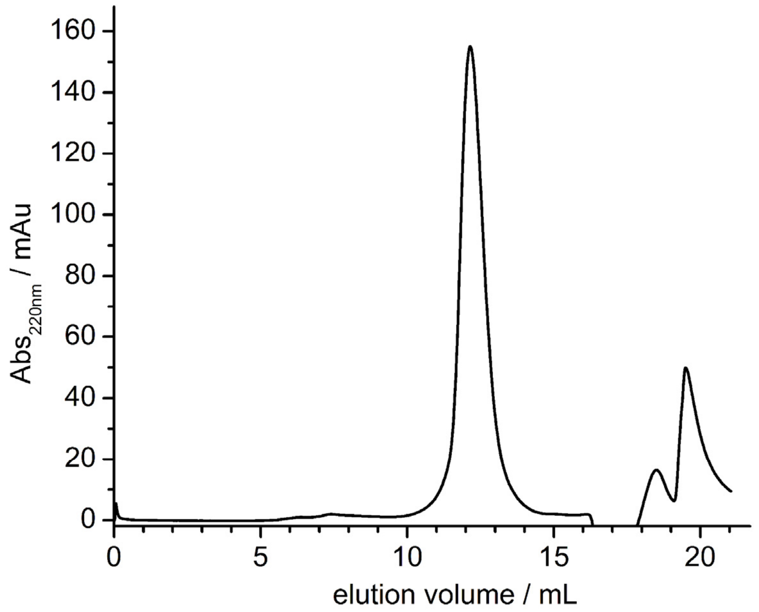

Figure S2. Chemical shift differences from [^1H,^1H]-TOCSY spectra for the backbone amide H^N (gray bars) and H^α (black bars) protons of residues 2-25 from cyc- and linear $Cd_2\gamma$-E_c-1.

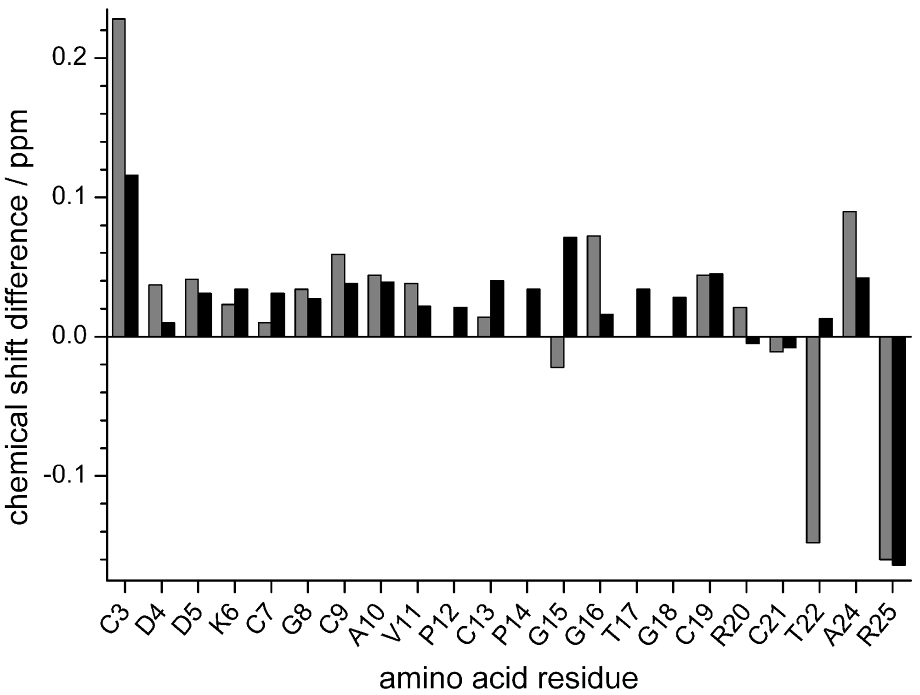

Table S1. Statistic of the 20 lowest energy structures for the two cysteine cluster arrangements (C3/C9, C21/C9), structure calculated without any cluster restraints (No MR) and Cys3/Cys9 structure calculated in assumption of both Pro residues being in trans conformation (C3/C9_T12T14).

Parameter	C3/C9	C21/C9	No MR	C3/C9_T12T14
XPLOR total energy	46.64 ± 0.69	171.48 ± 63.15	25.58 ± 0.29	62.91 ± 1.14
Quality of peak assignment (CYANA)	0.989 (1)	0.982 (10)	0.979 (11)	0.980 (10)
**Procheck-NMR (residues 2-23)**				
In most favored regions (%)	58,3	55	59.3	59.3
In additional allowed (%)	41.7	45	40	40.7
In generously allowed (%)	0	0	0.7	0
In disallowed regions (%)	0	0	0	0
**Verify 3D [30] (residues 2-23)**				
Raw score	0.43	0.41	0.38	0.42
Z-score	−0.48	−0.80	−1.28	−0.64

* Numbers in brackets correspond to the quantity of non-used NOE restraints by CYANA.

Table S2. NOE statistic for circular and linear $Cd_2\gamma$-E_c-1 structures calculated with CYANA.

Parameter	cyc-$Cd_2\gamma$-E_c-1	lin-$Cd_2\gamma$-E_c-1
NOE restraints: Total	201	157
Sequential (|i-j| < 1)	142	103
Medium (1 < |i-j| < 5)	38	44
Long range (|i-j| < 5)	21	10
